# Bone Marrow Adipocytes: The Enigmatic Components of the Hematopoietic Stem Cell Niche

**DOI:** 10.3390/jcm8050707

**Published:** 2019-05-18

**Authors:** Vincent Cuminetti, Lorena Arranz

**Affiliations:** 1 Stem Cell Aging and Cancer Research Group, Department of Medical Biology, Faculty of Health Sciences, UiT – The Arctic University of Norway, 9019 Tromsø, Norway; vincent.cuminetti@uit.no; 2 Department of Hematology, University Hospital of North Norway, 9019 Tromsø, Norway; 3 Young Associate Investigator, Norwegian Center for Molecular Medicine (NCMM), University of Oslo, 0349 Oslo, Norway

**Keywords:** Bone marrow adipocytes, mesenchymal stromal cells, hematopoietic stem cells, hematopoietic stem cell niche, hematopoiesis, hematopoietic malignancies, metabolism

## Abstract

Bone marrow adipocytes (BMA) exert pleiotropic roles beyond mere lipid storage and filling of bone marrow (BM) empty spaces, and we are only now beginning to understand their regulatory traits and versatility. BMA arise from the differentiation of BM mesenchymal stromal cells, but they seem to be a heterogeneous population with distinct metabolisms, lipid compositions, secretory properties and functional responses, depending on their location in the BM. BMA also show remarkable differences among species and between genders, they progressively replace the hematopoietic BM throughout aging, and play roles in a range of pathological conditions such as obesity, diabetes and anorexia. They are a crucial component of the BM microenvironment that regulates hematopoiesis, through mechanisms largely unknown. Previously considered as negative regulators of hematopoietic stem cell function, recent data demonstrate their positive support for hematopoietic stem cells depending on the experimental approach. Here, we further discuss current knowledge on the role of BMA in hematological malignancies. Early hints suggest that BMA may provide a suitable metabolic niche for the malignant growth of leukemic stem cells, and protect them from chemotherapy. Future in vivo functional work and improved isolation methods will enable determining the true essence of this elusive BM hematopoietic stem cell niche component, and confirm their roles in a range of diseases. This promising field may open new pathways for efficient therapeutic strategies to restore hematopoiesis, targeting BMA.

## 1. Introduction

Bone marrow adipocytes (BMA) were first seen in the 19th century, when Franz Ernst Christian Neumann observed red and yellow regions in the bone marrow (BM). One century later, adipocytes were identified as the major cell component of the yellow marrow [1]. In adults, BMA represent between 50 and 70 percent of the BM volume, and about 5 percent of whole body fat [2]. BMA are also present in the red marrow, in charge of the process of hematopoiesis or blood cell formation, including a variety of cell subsets such as erythrocytes, platelets and leukocytes, i.e., lymphocytes, monocytes and granulocytes [3]. These highly specialized and heterogeneous cell subsets arise from the differentiation of hematopoietic stem cells (HSC), located at the top of the blood system hierarchy [4]. HSC reside in particular microenvironments or niches in the BM, which are defined as the close environments in the vicinity of HSC with a variety of cell types and molecules that exert a fine-tuned regulation of HSC survival, self-renewal, differentiation and retention [5,6]. In the BM, HSC are found preferentially in perivascular niches, mostly sinusoids, formed by mainly endothelial cells and mesenchymal stromal cells (MSC) [7,8]. A smaller fraction of HSC localizes adjacent to small-diameter arterioles, adjacent to neural/glial antigen 2^+^ periarteriolar cells and MSC [7,9,10]. However, a variety of cell types have been involved in HSC regulation, including BMA [11]. Interestingly, although BMA have been traditionally considered as negative regulators of the HSC niche [11], more recent data point to BMA as key players during hematopoietic regeneration [12].

BMA are heterogeneous in terms of development, size, numbers, lipid composition, genomic expression and localization [13]. Heterogeneity depends on factors such as species, mouse strain [13] and gender [14]. Accumulation of BMA relates to pathogenesis in several disorders such as osteoporosis [15,16] and type II diabetes [17]. In this scenario, the purpose of this review is to integrate the current knowledge on BMA physiological functions and their involvement in disease mechanisms, with a focus on identifying gaps in the literature and generating interest in BMA potential as targets of future therapeutic strategies.

## 2. BMA: Human versus Mouse Features

During the 1980s, development of magnetic resonance imaging (MRI) and its use to study BM disorders allowed better characterization of BMA distribution [18]. In humans, at birth, the marrow is fully hematopoietic and contains no adipocytes. Early in life, a process of conversion of hematopoietic to fatty marrow takes place from distal towards central skeleton that continues throughout aging [19]. In long bones, tissue replacement starts in diaphysis with relative preservation of metaphyseal hematopoiesis. In the femur diaphysis, for example, conversion happens in 10 year-old children, followed by the distal metaphysis after 20 years. Femoral BM adiposity has an adult pattern after 24 years [20]. At the end of adolescence, hematopoietic marrow remains in the proximal metaphysis of femur and humerus as well as in spine, sternum, ribs, and skull. BMA arise later in the axial skeleton along the central axis, but in adults, they are present in the red marrow of sternum, ribs, pelvis and vertebral bones [21]. In the spine, BM adiposity grows from 27% to 70% in individuals between 10 and 76 years of age [22]. The rate of conversion may vary in other areas such as sacrum and BMA expansion towards lumbar vertebrae [22,23]. In contrast, the heel does not correlate with aging, as it contains already around 83% BMA in 10 year-old children, indicative of the much earlier onset of conversion in peripheral skeleton [22]. However, the process may be reverted in the extremities at any age in case of sustained increase in demand of hematopoiesis [22].

Rodents have lower BM adiposity than humans, but their BMA expansion follows similar centripetal pattern. Different types of BMA were already observed 40 years ago by Mehdi Tavassoli, when he suggested that adipocytes from red and yellow marrow are differentially affected by hemolysis and have distinct lipid composition [24,25]. These observations where recently confirmed when Scheller et al. defined two groups of adipocytes that differ in their location and response to physio-pathological conditions [13]. Constitutive BM adipose tissue (cBMAT) localizes in distal tibia and caudal vertebrae. These adipocytes develop very early after birth in the yellow marrow, occupy most of the BM cavity and are similar to adipocytes in white adipose tissue. In the proximal tibia near fibula junction, femur and axial skeleton, BMAT develops later, it is found as single or clustered smaller adipocytes interspersed with hematopoietic cells, and it responds to a range of nutritional, environmental, genetic, and endocrine factors. This is the regulated BMAT (rBMAT). Adipocytes from rBMAT reduce size and number after for example three weeks of cold exposition [13], fasting [26] or prolonged exercise [27], but also during lactation [28] or hematological malignancy such as acute myeloid leukemia (AML) [29]. Conversely, conditions that increase size and number of rBMAT include aging, high fat diet, caloric restriction and anorexia, irradiation, or treatments with hypoglycemic thiazolidinediones (insulin-mimetic drugs used for type 2 diabetes treatment) and hyperglycemic glucocorticoids [17,30]. Thus, seemingly opposed conditions from the metabolic perspective result in similar rBMAT expansion. Importantly, characterization of BMA subtypes in humans is yet to be done.

## 3. Sources of BMA

BMA develop from MSC differentiation in the BM, which can also differentiate into chondroblasts and osteoblasts [31]. MSC were functionally defined by the International Society of Cellular Therapy as plastic-adherent, they show expression of CD105 CD73 CD90, and lack expression of CD45 CD34, CD14 or CD11b, CD79α or CD19, and HLA-DR surface molecules, and must have the ability to differentiate into osteoblasts, adipocytes and chondroblasts in vitro [32]. Thus, MSC functionality has mainly been studied in vitro, so the adipocyte lineage in the BM is not completely clear yet, particularly in humans.

**Figure 1:**
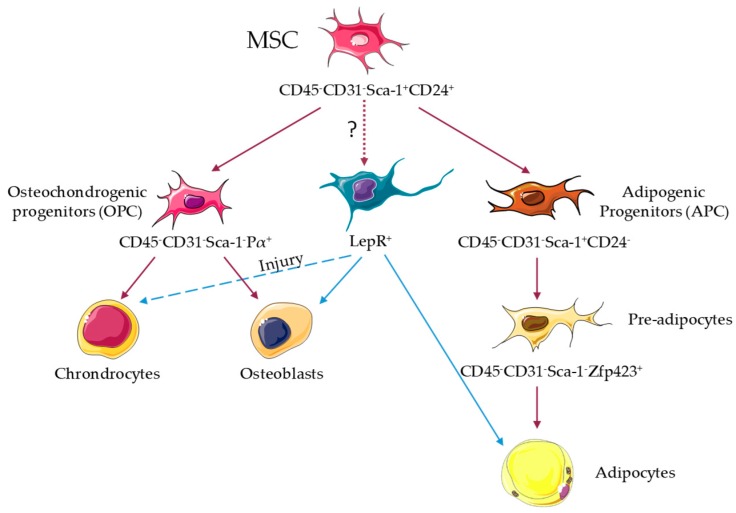
Adipocyte lineage in the mouse bone marrow. Stem cell-like mesenchymal stromal cells (MSC) capable of in vivo tri-lineage differentiation in the bone marrow are immunophenotypically defined as CD45^−^ CD31^−^ Sca-1^+^ CD24^+^, and show overlap with leptin receptor-cre^+^ (LepR^+^), Pdgfr-α(Pα)^+^ Sca-1^+^, and nestin^+^ cells. MSC give rise to osteochondrogenic progenitors (OPC) CD45^−^ CD31^−^ Pα^+^ Sca-1^−^ and adipogenic progenitors (APC) CD45^−^ CD31^−^ Sca-1^+^ CD24^−^. APC differentiate in CD45^−^ CD31^−^ Sca-1^−^ Zfp423^+^ pre-adipocytes that give rise to adipocytes [38]. LepR^+^ cells are able to differentiate into adipocytes and osteoblasts in vivo, but their ability to differentiate into chondrocytes was only observed upon injury [34].

In mice, MSC in neonatal BM develop during embryogenesis from Osterix1-expressing cells [33], and from leptin receptor-expressing cells in adult BM [34]. In adult mice, MSC have been defined by their ability of tri-lineage differentiation in vitro, activity of colony forming unit fibroblast, lack of expression of CD45 CD31 Ter119, and showing expression of different markers such as leptin receptor, Pdgfr-α Sca-1, or nestin [34,35,36,37]; all of them at least partially overlapping in their identities. Recent lineage-tracing studies have shed further light, revealing that MSC capable of tri-lineage differentiation in vivo are immunophenotypically defined as CD45^−^ CD31^−^ Sca-1^+^ CD24^+^, and also overlap with the above [38]. Leptin receptor-cre^+^ cells are able to differentiate into adipocytes and osteoblasts in vivo, but their ability to differentiate into chondrocytes is only revealed upon injury [34] (Figure 1). Further, nearly all leptin receptor-cre^+^ cells express Pdgfr-α, and 12% express Sca-1 [34]. Recent single-cell transcriptomic profiling of leptin receptor-cre^+^ cells shows the heterogeneity of this compartment, and has identified four different clusters; *Mgp*^high^ and especially *Lpl*^high^ express adipogenesis-associated markers, and *Wif1*^high^ and *Spp1*^high^*Ibsp*^high^ are osteo-primed [39]. Adipogenic leptin receptor-cre^+^ clusters can be distinguished by ESM1 staining, while osteogenic leptin receptor-cre^+^ clusters express CD200 and CD63 [39]. Conversely, CD45^−^ CD31^−^ Sca1^+^ CD24^+^ multipotent cells express high levels of *Lepr*, *Scf* and *Vcam1*, suggesting that they may represent a further purification step of MSC [38] (Figure 1). CD45^−^ CD31^−^ Sca1^+^ CD24^+^ MSC give rise to CD45^−^ CD31^−^ Sca-1^−^ Pdgfr-α^+^ osteochondrogenic progenitors and CD45^−^ CD31^−^ Sca-1^+^ CD24^−^ adipose progenitors, and the latter differentiate into adipocyte precursors CD45^−^ CD31^−^ Sca-1^−^ Zfp423^+^ [38] (Figure 1). However, no efforts were taken to uncover the potential different origins for cBMA and rBMA. Future work will be required for a complete understanding of mesenchymal hierarchy, from stem cells through multipotent and oligopotent progenitors to fully differentiated cells. Single-cell transcriptome analyses together with in vivo lineage tracing and fate mapping through barcoding will open new avenues in this field.

Interestingly, aging is linked to increased content of BMA in both human and mice [40], and also biased differentiation of MSC towards adipocyte lineage in mice [38]. Although increased secretion of dipeptidyl peptidase-4 (Dpp4) by adipogenic cell populations including MSC after adipogenic differentiation, has been related to reduced bone healing in old mice, Dpp4 did not affect adipogenic differentiation [38]. Future studies should focus on understanding the underlying mechanisms of age-related adipogenic biased MSC differentiation, and confirm the validity of this process in humans.

Recent data suggest that BM stromal cells could also be a source of adipocyte progenitors for other fat pads, and that BM-derived cells contribute to around 10% of cells in these sites [41]. Although these experiments were performed under BM transplantation, they point to a potential BM origin for some extramedullary adipocytes under steady-state conditions [42,43]. Future work is needed to understand the mechanisms underlying adipocyte progenitor egress from BM and homing into extramedullary sites. Adipocytes have a range of functions in the organism, such as contributing to wound healing after skin injury in mice, where they regenerate from myofibroblasts in hair follicles [44]. Moreover, adipocytes were recently reported to be motile in *Drosophilia melanogaster*, and contribute to wound healing through peristaltic mechanisms [45]. Further investigations are required to understand these phenomena better, particularly in mammals, but these data raise interest in the potential role of BMA and their progenitors in extramedullary hematopoiesis and homeostasis.

## 4. BMA Metabolic Function

The well-known metabolic role of adipocytes in the organism is storage and release of lipids depending on the energetic status. When energy intake is higher than expenses, adipocytes store fatty acids (FA) as triglycerides under the control of insulin. When energy is required, catabolic hormones such as adrenaline, glucocorticoids, and glucagon stimulate hydrolysis of triglycerides into FA and glycerol [46]. The first characterization of lipids in the BM was made by Mehdi Tavassoli, who observed that the red marrow preferentially contains myristic and palmitic acid (saturated FA) while the yellow marrow preferentially contains myristoleic and palmytoleic acid (mono-unsaturated FA) [25]. Comparison of lipid content in subcutaneous adipose tissue and BMA revealed that BMA contained less mono-unsaturated FA and more saturated FA. Further characterization showed that BMA from proximal femur had more saturated FA than BMA from proximal tibia [47]. These observations emphasize the metabolic heterogeneity of BMA that should be the subject of future research in the context of their regulatory activities in the BM.

Insulin and thiazolidinedione stimulate the expression of genes related to lipid metabolism in BMA such as FA synthase, FA binding protein, hormone-sensitive lipase and FA translocase. Interestingly, they do not induce translocation or expression of the insulin-sensible glucose transporter Glut-4, suggesting that BMA metabolism is based on lipid rather than glucose metabolism [48]. Further, while BMA have a lipid metabolism similar to classic adipocytes from white adipose tissue [49], they also seem to express specific markers of brown adipocytes such as UCP-1, PGC-1α, Prdm16, FoxC2 and β3-adrenergic receptor [50]. However, these markers are between 10,000 and 20,000 times less expressed in BMA than in brown adipocytes from brown adipose tissues [51]. This may suggest that BMA have a mixed metabolic phenotype between white and brown adipocytes; i.e., beige adipocytes. Nevertheless, it may also be reflection of BMA heterogeneity, meaning that rBMA that respond to cold exposition could have a brown-like phenotype under normal conditions while cBMA could be more similar to white adipocytes [14]. In this regard, thorough characterization of distinct immunophenotypical profiles for both types of BMA will be highly valuable in the hematopoietic field.

Surprisingly, while rBMAT expand with aging and high fat diet [17,30], brown adipocyte markers in BMA decrease with aging and diabetes, suggesting a link between BMA metabolic function and physio-pathological conditions [50]. Aging and obesity are associated with functional impairment of adipose tissue metabolism [52]. Accumulation of monocyte-macrophages in adipose tissue of obese individuals is associated with low-grade chronic inflammation and development of diabetes [53,54]. At least partially, these monocytes are recruited from BM through leptin receptor signaling, as chimeric wild-type (WT) mice reconstituted with leptin receptor-deficient BM and challenged with high-fat diet had lower body weight, adiposity, and inflammation, together with greater insulin sensitivity, compared with WT mice reconstituted with WT BM [55]. The potential direct cross-talk between monocytes and BMA in the BM is yet to be elucidated. However, a shift of MSC differentiation to the adipogenic lineage is found in diabetes and under chronic use of steroids and thiazolidinediones [56], and mature BMA stimulate BM MSC differentiation into new fat via secretion of MCP-1 [57], thereby potentially influencing hematopoietic output. Further, BMA are negative regulators of hematopoiesis under normal conditions [11] so future studies on the interplay between HSC and BMA during aging and diabetes will be highly relevant.

Intriguingly, inverse conditions to diabetes and high fat diet such as caloric restriction and anorexia nervosa are also associated with BMA increase [58,59]. This seemingly contradiction could be explained by the fact that the energetic cost of maintaining hematopoiesis by HSC, and producing billions of blood cells daily, is higher than adipogenesis done by MSC. So, when energy is limited, hematopoiesis slows down and hematopoietic cells are rapidly replaced by BMA with preservation of tissue architecture; situation that can be restored fast when conditions change back to normal. In addition, BMA could be understood as a fine-tuned regulated expandable-contractible fat storage in the BM serving to minimize systemic energy requirements for sustaining optimal hematopoiesis [60]. The underlying mechanisms are not completely understood but c-kit signaling has been suggested to couple MSC and HSC differentiation in BM. In this regard, loss of function mutations in the c-kit receptor and membrane-bound kit-ligand result in certain hematopoietic defects such as anemia and mast cell depletion, and absence of BMA in long bones and lumbar vertebrae [61].

During energetic demand by the organism, lipid mobilization from adipose tissue is stimulated by adrenergic signals from the sympathetic nervous system through β-adrenergic receptors (βAR) [62]. Unlike adipose tissue, BMA exhibit little catabolic response to βAR stimulation, although resistance is more pronounced in distal regions of the skeleton where BMA participation in hematopoiesis is less evident [26]. Despite recent data showing that BM MSC can be a source of beige adipocytes for white adipose tissue induced by β3AR activation [63], local responses in BM remain elusive. Interestingly, cyclical β3AR stimulation in BM MSC inhibits their expression of Cxcl12, which results in circadian egress of HSC and progenitors from BM [64]. In addition, damage to the microenvironment with loss of the neural-MSC regulatory circuit results in reduction of Cxcl12 that then allows expansion of mutant HSC in myeloproliferative neoplasms [65]. In this scenario, understanding the coordinated effect of β3AR activation on BMA seems timely.

Additional signals might be involved in the regulation of BMA metabolism. Metabolites such as lactate [66] and succinate [67] seem to be important players in the activation of brown adipocytes in brown and white fat pads. Succinate is product of the tricarboxylic acid cycle, and interestingly, its accumulation in brown adipocytes in response to cold occurs independently of AR activation. Further, oxidation of succinate by succinate dehydrogenase initiates production of reactive oxygen species, and drives thermogenic respiration in those cells. Pharmacological elevation of circulating succinate drives UCP1-dependent thermogenesis by brown adipose tissue in vivo, protecting against diet-induced obesity and improving glucose tolerance [67]. This points to succinate as a promising therapeutic target. However, succinate is increased 24-fold in BM stromal cells derived from diabetic mice compared to normoglycaemic mice [68]. In vitro, extracellular succinate binds to its receptor in osteoclastic lineage cells and stimulates osteoclast differentiation, a process that may influence the bone resorption seen in vivo in diabetic mice [68]. Unfortunately, the potential involvement of BMA was not studied. Succinate is also defined as an oncometabolite, with important roles in cancer, inflammation and hematopoiesis through both cell- and non-cell-autonomous mechanisms [69]. Thus, integrative studies on the role of succinate in hematopoiesis with potential participation of BMA will be highly relevant.

## 5. Sexual Dimorphism in BMA

The use of MRI to study BM adiposity also revealed differences between men and women. A first study showed that men between 17 and 42 years of age had higher amount of BMA in the sacrum compared with women [23]. This observation was confirmed in a larger study with 154 healthy subjects, that showed that the most prominent difference (12%) in marrow adiposity between men and women occurs from 31 to 50 years of age [70]. However, beyond 50 year-old individuals, the difference is reversed and menopausal women have 10% more BMA than age-matched men [71]. Interestingly, estrogen-supplemented post-menopausal women keep lower BMA content, suggesting a negative role for estrogens in BMA development [72].

These observations make sense considering that sexual dimorphism in white adipose tissue in rats was observed almost 60 years ago [73]. Moreover, in rats, estrogens are negative regulators of adipogenesis and central abdominal fat accumulation at least partially by inhibiting S100A6 expression [74]. Human sexual dimorphism in adipose tissue development has been extensively studied in the context of metabolic diseases. Women have more subcutaneous fat than men, who have more visceral fat. In obesity, men exhibit “apple-shape” android obesity, which is more deleterious metabolically than women “pear-shape” gynoid obesity [75,76]. Visceral adipose tissue is more deleterious metabolically due to higher lipolysis rate, lower insulin-sensitivity and portal vein drainage of adipokines and FA towards the liver, resulting in production of inflammatory mediators such as C-reactive protein and hepatotoxicity [77], but also in lipotoxicity of these FA in other insulin-sentive organs, resulting in insulin-resistance [78]. Based on these observations, it is reasonable to hypothesize that BMA are negatively regulated by estrogens in women previous to menopause, and BMA could be similar to visceral adipocytes, but no research group has tried to compare BMA with adipocytes located in different fat pads to date. Future work should confirm the validity of these ideas. However, BMA size and number correlate with total body fat in premenopausal obese woman [79], suggesting additional regulatory factors upstream of estrogens that should be subject of future work in the context of obesity.

There is no general consensus on BMA sexual dimorphism in rodents, as marrow adiposity varies between strains and most of the time only young animals are used in experiments. For example, C57BL/6J mice accumulate rBMAT in proximal tibia later than C3H/HeJ mice [13]. However, the content of cBMAT in tibia is similar between the females of both strains and, unlike women, female mice have higher rBMAT content than males [13,14]. Marrow adiposity in rodents is regulated by estrogens. Estrogen deficiency in ovariectomized females leads to higher content of rBMAT, with little effect on cBMAT [14,80]. Conversely, estrogen supplementation decreases marrow adiposity [81].

**Figure 2:**
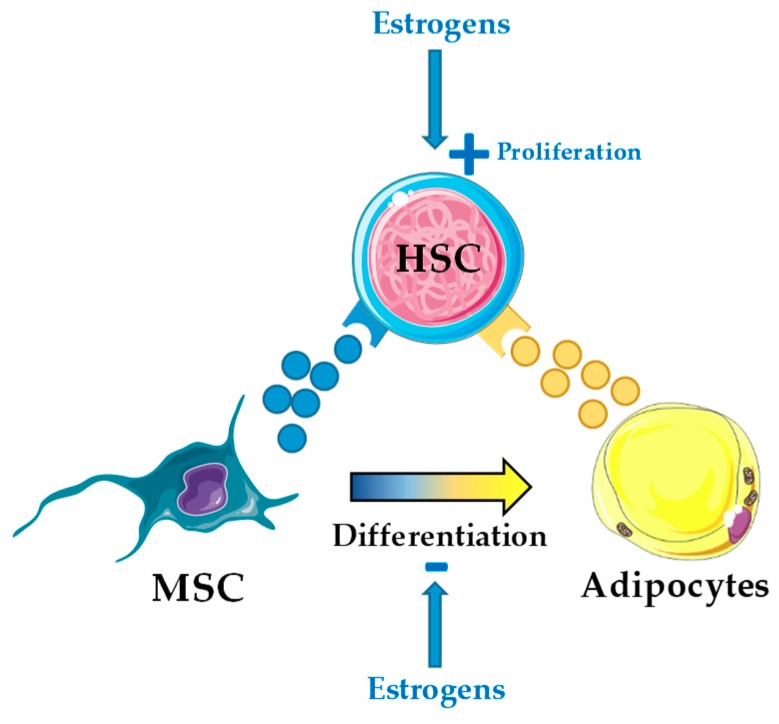
Estrogens may exert both cell- and non-cell-autonomous effect on hematopoietic stem cells (HSC). Estrogens directly regulate HSC self-renewal and proliferation through estrogen receptor (ER)-α activation in HSC [82,83], but potentially also indirectly through the bone marrow HSC niche by inhibition of mesenchymal stromal cell (MSC) differentiation into adipocytes.

In mice, estrogens are directly involved in hematopoiesis and promote HSC self-renewal through cell-autonomous mechanisms via activation of the α-isoform of the estrogen receptor [82]. Activation of this receptor with tamoxifen, an estrogen analog, induced apoptosis in short-term HSC and multipotent progenitors, and promoted proliferation of long-term HSC [83]. Further, tamoxifen blocked development of myeloproliferative neoplasm in a JAK2-V617F^+^-induced mouse model and enhanced chemotherapy efficiency in an MLL-AF9^+^-induced mouse model of AML [83]. In this scenario, understanding the coordinated action of estrogens in hematopoiesis both cell-autonomous on HSC and non-cell-autonomous through BMA seems timely (Figure 2).

The role of testosterone regulating BMA content is unknown. Testosterone level correlates inversely with obesity, but the cause seems to be expression of aromatase by adipose tissue, which converts testosterone in estradiol in obese men [84]. Estradiol then exerts an inhibitory loop on the hypothalamic-pituitary axis that inhibits testosterone production. Moreover, leptin, the circulating levels of which are increased in obesity, further inhibits testosterone production by Leydig cells [84]. The decrease in testosterone in obese men may be a risk factor in the context of metabolic syndrome, as low testosterone correlates with insulin resistance, but the causal effect mechanism, if any, remains unknown [85]. Recent data from in vitro experiments suggest that testosterone controls adipocyte differentiation and metabolism [86,87], but its effect in BMA is yet to be elucidated.

## 6. Role of BMA in Hematopoiesis: BMA as Components of the BM HSC Niche

Like any other adult stem cell, HSC are found in specialized microenvironments called niches [7]. Richard Schofield proposed the concept of the niche in 1978, defined as the close environment of stem cells, regulating their survival, retention, differentiation and self-renewal by a combination of soluble factors and cell contact mechanisms [6]. Extensive work has been performed in the last 20 years to characterize the HSC niche. The team of David Scadden originally described that osteoblasts expressing Notch ligand jagged 1 support increased numbers of HSC through activation of Notch1 in vivo [88]. The same year, osteoblasts were shown to maintain HSC anchored in the marrow by expression of N-cadherin in osteoblasts and β-catenin in long-term HSC [89]. Later, endothelial cells from sinusoids were found to host most HSC in the BM [90]. The HSC niche in the BM is now better understood, and perisinusoidal niches formed by mainly MSC and endothelial cells contain the majority (85%) of HSC [8,91] and support HSC by major production of Scf and Cxcl12 [92,93]. The endosteal niche contains progenitors of the lymphoid lineage [94], though recent data locate common lymphoid progenitors and pro-B cells near MSC and sinusoids as well [95,96].

MSC are an essential cell component of the vascular niche, and they were originally described as a major source of Cxcl12, which maintains HSC in the niche [93,97]. MSC have been further characterized in vivo by use of mouse models to help trace them, i.e., leptin receptor-cre^+^ cells and nestin-gfp^+^ cells [35,36,37], or by prospective identification, isolation and transplantation, i.e., Pdgfr-α^+^ Sca-1^+^ cells [37]. As mentioned previously, all of these cell types are partially overlapping. Leptin receptor-cre^+^ MSC labels a population of perivascular cells that are the main source of Cxcl12 and Scf in the BM, and are crucial for the maintenance of HSC in the vascular niche [92,94,98]. In addition to their HSC regulatory activity, leptin receptor-cre^+^ MSC differentiate into osteoblasts and adipocytes in vivo [12,34], suggesting a main contribution in bone remodeling and turn-over of additional cell components of the niche, such as BMA. Further, the recently identified MSC population capable of tri-lineage differentiation in vivo, CD45^−^ CD31^−^ Sca-1^+^ CD24^+^, was found to largely overlap with leptin receptor-cre^+^ cells and Pdgfr-α^+^ Sca-1^+^ cells, and express high levels of Scf [38].

**Figure 3:**
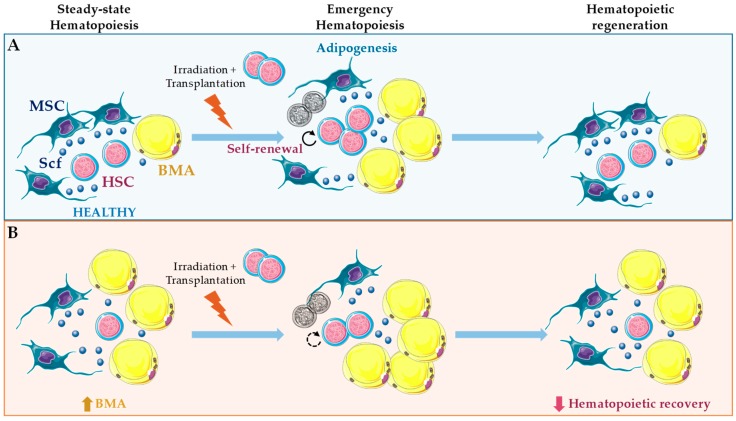
Role of bone marrow adipocytes (BMA) in hematopoietic regeneration. (**A**) In long bones, irradiation and chemotherapy promote adipogenesis in a subset of leptin receptor-cre^+^ mesenchymal stromal cells (MSC) traced by Adipoq-cre. Stem cell factor (Scf) from leptin receptor-cre^+^ MSC and BMA is essential to support hematopoietic stem cell (HSC) regeneration under those conditions [12]. (**B**) Under steady-state hematopoiesis, Scf from BMA promotes HSC maintenance only in bones where these cells are abundant, such as caudal vertebrae. However, here, BMA have a negative impact on hematopoietic regeneration [12]. Further, unbalanced numbers of BMA through intratibial transplantation followed by irradiation inhibits hematopoietic regeneration. Using this approach, only transplantation of multipotent CD45^−^ CD31^−^ Sca-1^+^ CD24^+^ promotes hematopoietic recovery [38].

**Figure 4:**
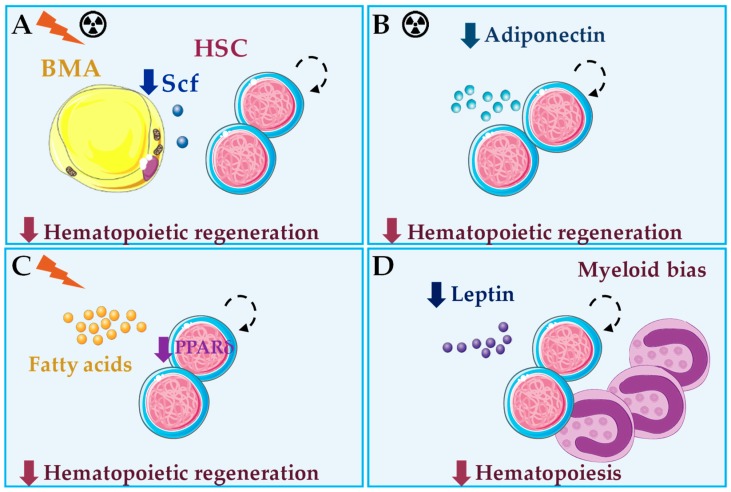
Summary of bone marrow adipocyte (BMA) roles in healthy hematopoiesis in vivo. (**A**) After irradiation or chemotherapy, Stem cell factor (Scf) produced by BMA is essential for hematopoietic regeneration [12]. (**B**) Adiponectin deficiency results in impaired hematopoietic regeneration after chemotherapy [103]. (**C**) Loss of the fatty acid oxidation regulator PPARδ leads to impaired hematopoietic regeneration after irradiation, through impaired asymmetric cell division of hematopoietic stem cells (HSC) [107]. (**D**) Under steady-state hematopoiesis, leptin or leptin signaling deficiency result in impaired hematopoiesis and myeloid skewing, through indirect mechanisms [108,109,110]. BMA produce and secrete adiponectin, fatty acids and leptin, but their roles in the hematopoietic effects described in **B**–**D** remain to be seen.

Conversely, in adult mice and humans, BMA correlate negatively with HSC function [11,99,100]. This negative correlation has been interpreted as a negative role of BMA in hematopoietic regulation, but almost 30 years ago BMA number was reported to increase after sublethal irradiation concomitantly with HSC proliferation [101]. HSC are reduced in adipocyte-rich vertebrae of the mouse tail compared to the adipocyte-free vertebrae of the thorax, and accelerated engraftment following irradiation was found using pharmacological and genetic approaches of reduced adipogenesis [11]. Back then, no efforts were taken to understand the cross-talk interactions involved in these effects. Further, those approaches seem to have major short-comings and affect endothelial cells in the vascular niche, and more recently, BMA together with leptin receptor-cre^+^ MSC were demonstrated as the major sources of Scf after irradiation and essential for hematopoietic recovery [12] (Figure 3 and Figure 4). This observation suggests that BM adipogenesis after irradiation represents a fast and efficient response to promote emergency hematopoietic regeneration [12]. Nevertheless, intratibial transplantation of adipocytic lineage committed cells followed by irradiation and competitive repopulation assays inhibited hematopoietic recovery. Using this strategy, only the multipotent CD45^−^ CD31^−^ Sca-1^+^ CD24^+^ was capable of increased repopulation with donor-derived long-term HSC and short-term HSC [38] (Figure 3). Future work will be needed to fully understand these seemingly contradictory results. However, it is reasonable to hypothesize that BMA may be essential for emergency hematopoiesis, and still be deleterious for this process, when present in unbalanced numbers.

BMA secrete a variety of factors, and some of these have HSC regulatory activity [49]. In mice, adiponectin produced by BMA acts through its receptor on HSC and stimulates their proliferation and multipotency in vitro by activation of the p38 MAPK pathway [102]. HSC pre-treated with adiponectin have enhanced hematopoietic reconstitution potential after transplantation in lethally irradiated mice [102]. Conversely, adiponectin deficiency in mice is associated with defective hematopoietic regeneration after chemotherapy [103] (Figure 4). Data on adiponectin regulation in human hematopoiesis are scarce. Adiponectin is also produced by BMA, and it seems to inhibit growth of myelomonocytic progenitors [104] but stimulate myelopoiesis from HSC in vitro [105]. Further, recombinant adiponectin blocked adipocyte formation in long-term BM cultures and reduced differentiation of cloned stromal preadipocytes, through increased expression of cyclooxygenase-2 and release of prostaglandin E2 [106]. Interestingly, adiponectin inhibits B lymphopoiesis only in the presence of stromal cells through a similar mechanism [105]. Thus, adiponectin stands out as a good candidate that mediates communication between BMA and HSC.

**Figure 5:**
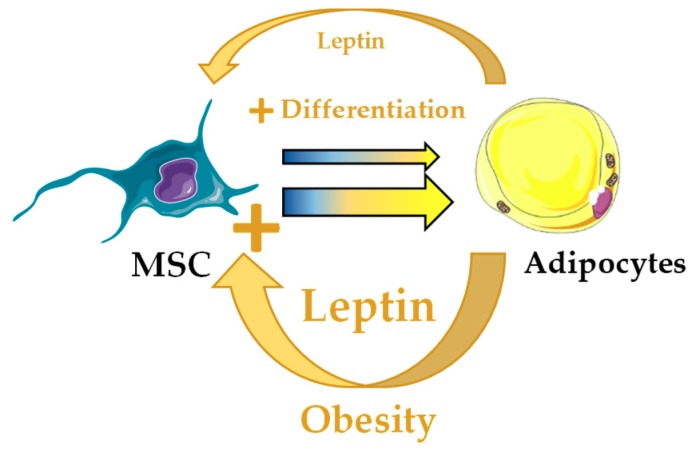
Potential alteration of the bone marrow hematopoietic stem cell niche through unbalanced leptin in obesity. Leptin produced by bone marrow adipocytes (BMA) stimulates adipocyte differentiation in leptin receptor-cre^+^ mesenchymal stromal cells (MSC). In obesity, increased leptin production by BMA would potentially bias MSC differentiation towards adipocyte lineage and result in hematopoietic abnormalities.

Another interesting candidate potentially involved in coordinating adipogenesis and hematopoiesis is leptin, but data are still controversial. Human BMA produce leptin in vitro [111]. Leptin is the main adipokine produced by white adipocytes in the organism and it has pleiotropic effects [112,113,114]. Leptin-deficient (ob/ob) mice show reduced number of circulating lymphocytes and increased monocytes, deficient erythrocyte production in spleen and inability to fully recover lymphopoietic populations after irradiation insult [115,116]. Further studies of ob/ob mice and leptin receptor-deficient (db/db) mice demonstrated impaired hematopoiesis, BM hypocellularity and myeloid skewing [108,109]. Interestingly, defects can be partially ameliorated when db/db mice are joined to WT partners in parabiosis, through exposure to healthy blood cells and endocrine factors whose identity is unknown [109,110] (Figure 4). Although several hematopoietic cell types, including HSC, have been reported to express the long form of leptin receptor, data on the role of leptin in direct regulation of hematopoietic cell proliferation are contradictory [117]. In cultures of murine or human BM cells, human leptin failed to stimulate cell survival or proliferation [118]. However, another study in mice showed that leptin stimulates proliferation of murine myelocytic progenitor cells and synergizes with Scf to promote HSC colony formation in vitro [119]. Interestingly, leptin also promotes differentiation of leptin receptor-cre^+^ MSC towards adipose lineage at the expense of the osteoblastic lineage in vivo [120]. Future work will be required for a better understanding of the effects of leptin in hematopoietic regulation both cell- and non-cell-autonomously, especially in obesity where leptin production by adipocytes is increased (Figure 5).

Scarce, but exciting, data are available on the role of FA secreted by BMA in hematopoiesis. A prospective study of human BM CD133^+^ cells cultured in presence of EPO or G-CSF, to promote erythropoiesis or granulopoiesis respectively, showed that the majority of resulting cells coexpress CD13 and CD36, or FA translocase, and identified this population as a common progenitor for erythroid and myeloid lineages by colony formation ex vivo [121]. Despite expression of CD36 suggest that FA locally released by BMA may be important for myelo/erythoid differentiation of hematopoietic progenitors, these are able to form colonies in the absence of FA. In mouse models, FA oxidation is essential for HSC maintenance, and loss of PPARδ (Peroxisome proliferator-activating receptor δ), important regulator of FA oxidation that is activated by FA, or inhibition of mitochondrial FA oxidation, result in repopulation defects after transplantation [107]. Mechanistically, inhibition of FA oxidation resulted in symmetric HSC division, whereas PPAR-δ activation increased asymmetric cell division [107] (Figure 4). The role of BMA in these events should be the subject of future work.

In vitro, human and mouse BMA can secrete inflammatory cytokines, particularly IL-6 and more subtle levels of TNF-α [122,123]. Findings demonstrated that BMA promote HSC survival, supporting HSC proliferation and differentiation after 5 weeks of coculture. In addition, BMA were found to express CXCL12, IL-8, colony-stimulating factor 3 (CSF3), and leukemia inhibitory factor (LIF) at similar levels to primary human BM MSC, whereas IL-3 was higher in BMA [124]. Future work should elucidate the relative inflammatory cytokine production by BMA as compared to other major sources in BM, and their specific role in steady-state hematopoiesis in vivo.

Finally, BMA may regulate HSC via cell-to-cell contact mechanisms, but these are not clearly defined yet. In mice, preadipocytic cell lines and BM stromal cells treated with troglitazone to induce adipogenesis, demonstrated enhanced HSC support in long-term serial dilution assays, compared to preosteoblastic cell lines and control-treated BM stromal cells, respectively [125]. In case of the preadipocytic cell line OP9, however, their ability to support primitive HSC is reduced when achieving complete adipocyte differentiation compared to their undifferentiated state or under osteogenic conditions. Preadipocytic OP9 cells display enhanced HSC support in cell-to-cell contact cultures compared to transwell, and multiple pathways seem to be involved, including Notch, Wnt, and Hedgehog [125]. In humans, BM MSC differentiated in vitro into adipocytes failed to maintain BM CD34^+^ hematopoietic progenitors in cell-to-cell cocultures, but induced their complete myeloid and B lymphoid differentiation [126]. Conversely, others showed that the relative amount of adipocytes, differentiated in vitro from BM MSC, supports CD34^+^ cell survival and inhibits their differentiation [127]. Others showed that BMA expanded in vitro as fibroblast-like fat cells inhibit granulopoiesis of CD34^+^ cells via neuropilin-1 through cell-to-cell contact mechanisms only, as this effect was not observed in transwell cocultures [128]. In vivo data are urgently needed to draw conclusions on the potential cell-to-cell communication between BMA and HSC, and whether this cross-talk may be bidirectional.

## 7. Role of BMA in Malignant Hematopoiesis

**Figure 6:**
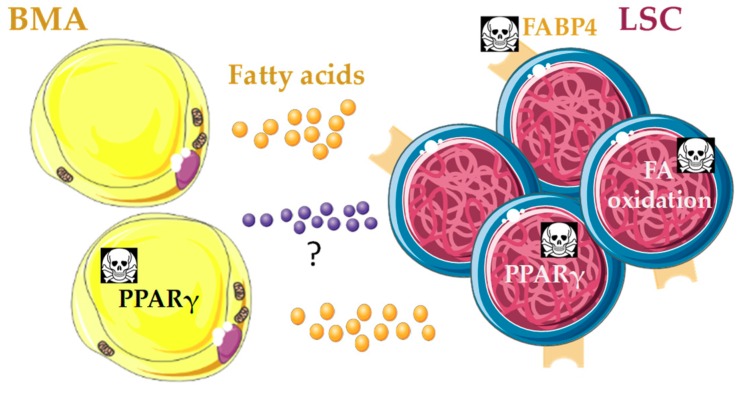
Potential bone marrow adipocyte (BMA) metabolic niche in vivo that promotes quiescence of leukemic stem cells (LSC) and protects them from chemotherapy. Leukemic cells depend on fatty acid (FA) oxidation as energy source, and BMA may fuel this requirement after rewire from LSC through unknown mechanisms. Several approaches with therapeutic potential go in this direction: genetic disruption of the FA transporter *FABP4* reduces leukemia bulk and promotes survival in acute myeloid leukemia (AML) mouse models [138,139]; pharmacological inhibition of FA oxidation induces LSC quiescence exit and sensitizes leukemia cells to chemotherapy in AML [140,141]; PPARγ agonists in combination with imatinib induce LSC quiescence exit, reduce stemness and promote apoptosis through reduction of *STAT5* expression, in human chronic myeloid leukemia [142]; and PPARγ agonists in in vivo xenograft models of AML induce BM adipogenesis, which rescues healthy hematopoiesis and blocks leukemic growth [29]. PPARγ agonists may improve lipid uptake and storage in BMA, and thereby disrupt FA supply to LSC.

Mutations in the stromal HSC microenvironment as sole alterations may drive myeloproliferative syndromes, myelodysplastic syndromes and secondary leukemia, juvenile myelomonocytic leukemia, and AML in mice [129,130,131,132]. These findings highlight the critical role played by the HSC niche in the initiation and development of hematological malignancies [133]. Besides, cancer cells shift their preferential metabolism from more efficient oxidative phosphorylation to glycolylis even under aerobic conditions, which is known as the Warburg effect [134]. The majority of cancer cells seem to be programmed to increase glucose uptake but reduce its oxidation in the Krebs cycle, thereby becoming more dependent on additional energy sources such as β-oxidation of FA [135,136]. Taken together, it is reasonable to hypothesize that BMA may play an active role in the generation of a metabolic niche suitable for maintenance of malignant blasts in the BM (Figure 6). This idea has already been demonstrated in gonadal adipose tissue, which fuels FA oxidation in CD36^+^ leukemic stem cells (LSC) and protects them from chemotherapy in a mouse model of blast crisis chronic myeloid leukemia (CML) [137].

However, data on the role of BMA in hematological malignancies are scarce. Human epidemiological data show that obesity, which correlates to higher BMA number and volume, is linked to higher frequency and mortality of leukemia [143]. Adipocytes differentiated from BM MSC in vitro promoted survival of acute myelomonocytic leukemia cell lines and primary cells in coculture, and this was linked to increased FA β-oxidation and upregulation of *PPARγ*, the FA transporter *FABP4*, *CD36*, and the antiapoptotic *BCL2* gene expression [144]. Further, genetic disruption of *FABP4* in AML cells or in mouse models blocked cell proliferation in vitro and induced leukemia regression in vivo (Figure 6). FABP4 was linked to more aggressive AML through increased *IL6* expression, resulting in *DNMT1* overexpression and silencing of *p15^INK4B^* tumor-suppressor gene in AML cells [138]. Others showed that the knockdown of *FABP4* increased survival in a HoxA9/Meis1-induced AML mouse model, and in vitro cocultures of adipocytes differentiated from BM MSC together with AML blasts showed activation of lipolysis and transfer of FA from adipocytes to AML blasts with participation of FABP4 [139] (Figure 6). This introduces the exciting concept that LSC and/or blasts could transform the BM adipocytic niche to meet the high demand of FA required to sustain their high proliferative rate [139]. In the same line, coculture of leukemic cell lines with adipocytes differentiated from BM MSC showed that leukemic cells induced morphological changes in adipocytes through secretion of growth differentiation factor 15, and as consequence of this remodeling adipocytes promoted leukemic cell growth [145].

Thus, despite limited data on BMA, FA metabolism is emerging as a promising strategy for the treatment of hematological malignancies. Pharmacological inhibition of FA oxidation decreased the number of quiescent leukemia progenitor cells in approximately 50% of primary human AML samples, sensitized leukemia cells to apoptosis induction by ABT-737 (mediated by proapoptotic Bcl-2 family members) and, when combined with either ABT-737 or cytosine arabinoside, had therapeutic effect in xenografts of AML cell lines in nude mice [140] (Figure 6). In human chronic lymphocytic leukemia (CLL), PPARα expression and activity increased by treatment with the synthetic glucocorticoid dexamethasone in vitro, and adipocyte-derived lipids, lipoproteins, and propionic acid conferred resistance to dexamethasone [141]. PPARα and FA oxidation enzyme inhibitors increased CLL cell death induced by dexamethasone in vitro and clearance of CLL xenografts in vivo. These findings suggest that FA oxidation is a mechanism of resistance to glucocorticoids in CLL [141].

These data should be considered with caution though, as another recent study showed that PPARγ agonist pioglitazone in combination with imatinib is efficient in making LSC exit quiescence to enter apoptosis in human BCR-ABL^+^ CML [142]. Pioglitazone reduces *STAT5* expression in LSC, thereby reducing the expression of key STAT5 target genes involved in regulation of quiescence and stemness, i.e., *HIF2α* and *CITED2* [142] (Figure 6). Thus, the molecular mechanism of the therapeutic effect of pioglitazone seems to be independent from its effects on FA metabolism, although this was not studied. Strikingly, pioglitazone was temporarily given to three CML patients in chronic residual disease despite continuous treatment with imatinib, and all of them achieved complete molecular remission, up to 4.7 years after withdrawal of pioglitazone [142]. Using both in vitro cocultures and in vivo xenograft models, another recent study showed that human AML disrupts the BM adipocytic niche, resulting in impaired myelo-erythroid maturation of healthy HSC. In vivo administration of PPARγ agonists such as GW1929 induced BM adipogenesis, which rescued healthy hematopoietic output and repressed leukemic growth [29] (Figure 6). The molecular mechanisms mediating communication between BMA and healthy or malignant HSC were not studied, nor was the potential contribution of FA transfer. Thiazolidinediones, such as pioglitazone, are PPARγ agonists that act as insulin-mimetics shifting the energy balance towards adipocyte storage, and are currently used as antidiabetic treatment [146]. Thus, it is reasonable to hypothesize that improvement in lipid uptake and storage from adipocytes contributes to disruption of FA supply to LSC, and that BMA play a key role in this process in the BM in vivo. Future work should test the validity of this exciting idea. Nevertheless, thiazolidinediones have substantial side effects that limit their clinical use. A recent study showed that genetic variations in human adipocytes derived from subcutaneous adipocyte precursors in vitro can predict patient antidiabetic response to rosiglitazone [147], and these will have to be considered in the assessment of the potential for thiazolidinediones as treatment against leukemia.

Additional mechanisms of BMA involvement in hematological malignancy progression may include secretion of mediators, such as TNF-α, IL-6, leptin and adiponectin [148]. Leptin activates its receptor on malignant cells from a range of myeloid and lymphoid neoplasms leading to stimulation of cell proliferation and cytokine secretion, and protection from apoptosis, through JAK-STAT, MAPK/ERK1/2, or PI3K signaling pathways [149]. However, the participation of BMA is unknown. In vitro experiments showed that adipocytes differentiated from BM MSC reduced sensitivity to doxorubicin and all-trans retinoic acid-induced apoptosis in primary acute promyelocytic leukemia cells. This antiapoptotic effect was dependent on cell-to-cell interactions but also leptin receptor signaling [150]. BMA are able to enhance proliferation and reduce apoptosis in coculture with multiple myeloma cells, but leptin contribution was minor [151]. Recently, in vivo blockade of leptin receptor signaling combined with invariant natural killer T cell stimulation resulted in higher therapeutic efficacy against multiple myeloma [152], which highlights the limitations of in vitro studies. The case of adiponectin is of particular interest as plasma adiponectin concentration correlates inversely with total fat mass [153], and low adiponectin is involved in diabetes [154]. Adiponectin activates protein kinase A, leading to decreased AKT activity and increased AMP-activated protein kinase (AMPK) activation. AMPK then induces cell cycle arrest and apoptosis in multiple myeloma cells, at least in part mediated by reduced expression of the enzyme acetyl-CoA-carboxylase, essential to lipogenesis [155]. Future work in vivo is required to understand the role of cytokines and adipokines secreted by BMA in hematological malignancies.

Considering that adipose tissue from extramedullary sites such as gonads protects LSC from chemotherapy in a mouse model of blast crisis CML [137], it is reasonable to think that BMA could exert this function in BM. In human xenografts of T-cell acute lymphoblastic leukemia (T-ALL) into NSG mice, T-ALL recovered from tail BM displays a non-cell-autonomous dormancy phenotype and higher intrinsic resistance to chemotherapy, compared to T-ALL from thorax vertebrae BM [156]. Those features are similar in T-ALL recovered from gonadal adipose tissue, suggesting that BMA may contribute to T-ALL hallmarks in tail BM [156]. Additional hints linking adipocytes to chemotherapy resistance include that obese mice transplanted with acute lymphoblastic leukemia (ALL) cells showed increased relapse after vincristine treatment, and lower levels of the drug in BM and blood [157,158]. Further, human and mouse ALL cell lines migrated towards conditioned media from adipocytes differentiated in vitro from MSC cell lines, in response to CXCL12, and mouse adipose tissue explants protected ALL cells against daunorubicin and vincristine [159]. Interestingly, mouse and human fat explants in vitro are capable of daunorubicin uptake, and further inactivation by expression of enzymes such as aldo-keto reductases and carbonyl reductases, which may contribute to the reduced BM levels of the drug 2 h after its injection [160]. Future studies must determine if these data stand for BMA as well. Of note, recently, chemotherapy-induced inhibition of adipogenesis has been suggested as a read-out of consolidation chemotherapy efficiency in AML patients during complete remission. Chemotherapy inhibited adipogenesis by promoting growth differentiation factor 15 secretion from BM mononucleated cells, thereby preventing relapse in AML patients [161].

## 8. Conclusion

BMA are a heterogeneous cell population that shows sexual dimorphism and changes with aging and a variety of physio-pathological conditions such as menopause, obesity, diabetes, anorexia nervosa and leukemia. BMA are currently accepted as HSC niche cellular components, but we are only beginning to understand the regulation exerted by BMA in steady-state hematopoiesis. Although BMA have been considered as negative HSC regulators for a decade, more recent data showed that BMA may be essential for emergency hematopoiesis or still be deleterious depending on the experimental approach. BMA heterogeneity may further underlie these seeming contradictions. Future research should focus on accurate in vivo lineage tracing, and immunophenotypical, metabolic, and functional characterization of cBMA and rBMA, in integrative studies on the healthy HSC niche.

Interestingly, most studies on the role of BMA in the HSC niche have focused on their potential support of HSC survival/proliferation, with little attention to the possible facilitation of HSC differentiation. However, myeloid skewing is a common event in obesity, diabetes, myeloid malignancies, and aging [162]; all conditions with increased BMA. Considering that extramedullary hematopoiesis in adipose tissue is preferentially axed towards the myeloid lineage [163,164], the involvement of BMA in such a process in the BM under health and disease should be subject of future work.

Finally, early hints suggest that LSC could transform BMA to provide a suitable metabolic niche for their malignant growth that also protects them from chemotherapy. However, most data on this topic are derived from adipocytes differentiated in vitro from BM MSC and cocultures with leukemic cells, which represents a major limitation and requires urgent in vivo confirmation. If this exciting idea is valid, then BMA could be seen as potential therapeutic targets to restore hematopoiesis in the context of malignancy. Indeed, pioneering studies showed therapeutic value for PPARγ agonists in CML patients and AML xenograft models, and BMA participated at least in the beneficial effects of these compounds against AML. However, the underlying molecular mechanisms remain largely unknown and need further investigation.

## References

[1] Zakaria E., Shafrir E. (1967). Yellow bone marrow as adipose tissue. Proc. Soc. Exp. Biol. Med..

[2] Hardouin P., Rharass T., Lucas S. (2016). Bone Marrow Adipose Tissue: To Be or Not To Be a Typical Adipose Tissue?. Front. Endocrinol..

[3] Ogawa M. (1993). Differentiation and proliferation of hematopoietic stem cells. Blood.

[4] Osawa M., Hanada K., Hamada H., Nakauchi H. (1996). Long-term lymphohematopoietic reconstitution by a single CD34-low/negative hematopoietic stem cell. Science.

[5] Dexter T.M., Allen T.D., Lajtha L.G. (1977). Conditions controlling the proliferation of haemopoietic stem cells in vitro. J. Cell. Physiol..

[6] Schofield R. (1978). The relationship between the spleen colony-forming cell and the haemopoietic stem cell. Blood Cells.

[7] Crane G.M., Jeffery E., Morrison S.J. (2017). Adult haematopoietic stem cell niches. Nat. Rev. Immunol..

[8] Morrison S.J., Scadden D.T. (2014). The bone marrow niche for haematopoietic stem cells. Nature.

[9] Kunisaki Y., Bruns I., Scheiermann C., Ahmed J., Pinho S., Zhang D., Mizoguchi T., Wei Q., Lucas D., Ito K. (2013). Arteriolar niches maintain haematopoietic stem cell quiescence. Nature.

[10] Itkin T., Gur-Cohen S., Spencer J.A., Schajnovitz A., Ramasamy S.K., Kusumbe A.P., Ledergor G., Jung Y., Milo I., Poulos M.G. (2016). Distinct bone marrow blood vessels differentially regulate haematopoiesis. Nature.

[11] Naveiras O., Nardi V., Wenzel P.L., Hauschka P.V., Fahey F., Daley G.Q. (2009). Bone-marrow adipocytes as negative regulators of the haematopoietic microenvironment. Nature.

[12] Zhou B.O., Yu H., Yue R., Zhao Z., Rios J.J., Naveiras O., Morrison S.J. (2017). Bone marrow adipocytes promote the regeneration of stem cells and haematopoiesis by secreting SCF. Nat. Cell Biol..

[13] Scheller E.L., Doucette C.R., Learman B.S., Cawthorn W.P., Khandaker S., Schell B., Wu B., Ding S.-Y., Bredella M.A., Fazeli P.K. (2015). Region-specific variation in the properties of skeletal adipocytes reveals regulated and constitutive marrow adipose tissues. Nat. Commun..

[14] Lecka-Czernik B., Stechschulte L.A., Czernik P.J., Sherman S.B., Huang S., Krings A. (2017). Marrow Adipose Tissue: Skeletal Location, Sexual Dimorphism, and Response to Sex Steroid Deficiency. Front. Endocrinol..

[15] Fazeli P.K., Horowitz M.C., MacDougald O.A., Scheller E.L., Rodeheffer M.S., Rosen C.J., Klibanski A. (2013). Marrow fat and bone--new perspectives. J. Clin. Endocrinol. Metab..

[16] Schwartz A.V. (2015). Marrow fat and bone: Review of clinical findings. Front. Endocrinol..

[17] Doucette C.R., Horowitz M.C., Berry R., MacDougald O.A., Anunciado-Koza R., Koza R.A., Rosen C.J. (2015). A High Fat Diet Increases Bone Marrow Adipose Tissue (MAT) But Does Not Alter Trabecular or Cortical Bone Mass in C57BL/6J Mice. J. Cell. Physiol..

[18] Mitchell D.G., Rao V.M., Dalinka M., Spritzer C.E., Axel L., Gefter W., Kricun M., Steinberg M.E., Kressel H.Y. (1986). Hematopoietic and fatty bone marrow distribution in the normal and ischemic hip: New observations with 1.5-T MR imaging. Radiology.

[19] Ricci C., Cova M., Kang Y.S., Yang A., Rahmouni A., Scott W.W., Zerhouni E.A. (1990). Normal age-related patterns of cellular and fatty bone marrow distribution in the axial skeleton: MR imaging study. Radiology.

[20] Moore S.G., Dawson K.L. (1990). Red and yellow marrow in the femur: Age-related changes in appearance at MR imaging. Radiology.

[21] Kricun M.E. (1985). Red-yellow marrow conversion: Its effect on the location of some solitary bone lesions. Skeletal Radiol..

[22] Liney G.P., Bernard C.P., Manton D.J., Turnbull L.W., Langton C.M. (2007). Age, gender, and skeletal variation in bone marrow composition: A preliminary study at 3.0 Tesla. J. Magn. Reson. Imaging.

[23] Duda S.H., Laniado M., Schick F., Strayle M., Claussen C.D. (1995). Normal bone marrow in the sacrum of young adults: Differences between the sexes seen on chemical-shift MR imaging. Am. J. Roentgenol..

[24] Tavassoli M. (1976). Marrow adipose cells. Histochemical identification of labile and stable components. Arch. Pathol. Lab. Med..

[25] Tavassoli M., Houchin D.N., Jacobs P. (1977). Fatty acid composition of adipose cells in red and yellow marrow: A possible determinant of haematopoietic potential. Scand. J. Haematol..

[26] Scheller E.L., Khandaker S., Learman B.S., Cawthorn W.P., Anderson L.M., Pham H.A., Robles H., Wang Z., Li Z., Parlee S.D. (2019). Bone marrow adipocytes resist lipolysis and remodeling in response to beta-adrenergic stimulation. Bone.

[27] Styner M., Pagnotti G.M., McGrath C., Wu X., Sen B., Uzer G., Xie Z., Zong X., Styner M.A., Rubin C.T. (2017). Exercise Decreases Marrow Adipose Tissue Through ss-Oxidation in Obese Running Mice. J. Bone Miner. Res..

[28] Bornstein S., Brown S.A., Le P.T., Wang X., DeMambro V., Horowitz M.C., MacDougald O., Baron R., Lotinun S., Karsenty G. (2014). FGF-21 and skeletal remodeling during and after lactation in C57BL/6J mice. Endocrinology.

[29] Boyd A.L., Reid J.C., Salci K.R., Aslostovar L., Benoit Y.D., Shapovalova Z., Nakanishi M., Porras D.P., Almakadi M., Campbell C.J.V. (2017). Acute myeloid leukaemia disrupts endogenous myelo-erythropoiesis by compromising the adipocyte bone marrow niche. Nat. Cell Biol..

[30] Devlin M.J., Rosen C.J. (2015). The bone-fat interface: Basic and clinical implications of marrow adiposity. Lancet Diabetes Endocrinol..

[31] Friedenstein A.J., Petrakova K.V., Kurolesova A.I., Frolova G.P. (1968). Heterotopic of bone marrow. Analysis of precursor cells for osteogenic and hematopoietic tissues. Transplantation.

[32] Dominici M., Le Blanc K., Mueller I., Slaper-Cortenbach I., Marini F., Krause D., Deans R., Keating A., Prockop D., Horwitz E. (2006). Minimal criteria for defining multipotent mesenchymal stromal cells. The International Society for Cellular Therapy position statement. Cytotherapy.

[33] Mizoguchi T., Pinho S., Ahmed J., Kunisaki Y., Hanoun M., Mendelson A., Ono N., Kronenberg H.M., Frenette P.S. (2014). Osterix marks distinct waves of primitive and definitive stromal progenitors during bone marrow development. Dev. Cell.

[34] Zhou B.O., Yue R., Murphy M.M., Peyer J.G., Morrison S.J. (2014). Leptin-receptor-expressing mesenchymal stromal cells represent the main source of bone formed by adult bone marrow. Cell Stem Cell.

[35] Bernal A., Arranz L. (2018). Nestin-expressing progenitor cells: Function, identity and therapeutic implications. Cell Mol. Life Sci..

[36] Mendez-Ferrer S., Michurina T.V., Ferraro F., Mazloom A.R., Macarthur B.D., Lira S.A., Scadden D.T., Ma’ayan A., Enikolopov G.N., Frenette P.S. (2010). Mesenchymal and haematopoietic stem cells form a unique bone marrow niche. Nature.

[37] Morikawa S., Mabuchi Y., Kubota Y., Nagai Y., Niibe K., Hiratsu E., Suzuki S., Miyauchi-Hara C., Nagoshi N., Sunabori T. (2009). Prospective identification, isolation, and systemic transplantation of multipotent mesenchymal stem cells in murine bone marrow. J. Exp. Med..

[38] Ambrosi T.H., Scialdone A., Graja A., Gohlke S., Jank A.M., Bocian C., Woelk L., Fan H., Logan D.W., Schurmann A. (2017). Adipocyte Accumulation in the Bone Marrow during Obesity and Aging Impairs Stem Cell-Based Hematopoietic and Bone Regeneration. Cell Stem Cell.

[39] Tikhonova A.N., Dolgalev I., Hu H., Sivaraj K.K., Hoxha E., Cuesta-Dominguez A., Pinho S., Akhmetzyanova I., Gao J., Witkowski M. (2019). The bone marrow microenvironment at single-cell resolution. Nature.

[40] Li Z., Hardij J., Bagchi D.P., Scheller E.L., MacDougald O.A. (2018). Development, regulation, metabolism and function of bone marrow adipose tissues. Bone.

[41] Arner P., Ryden M. (2017). The contribution of bone marrow-derived cells to the human adipocyte pool. Adipocyte.

[42] Crossno J.T., Majka S.M., Grazia T., Gill R.G., Klemm D.J. (2006). Rosiglitazone promotes development of a novel adipocyte population from bone marrow-derived circulating progenitor cells. J. Clin. Invest..

[43] Majka S.M., Fox K.E., Psilas J.C., Helm K.M., Childs C.R., Acosta A.S., Janssen R.C., Friedman J.E., Woessner B.T., Shade T.R. (2010). De novo generation of white adipocytes from the myeloid lineage via mesenchymal intermediates is age, adipose depot, and gender specific. Proc. Natl. Acad. Sci. USA.

[44] Plikus M.V., Guerrero-Juarez C.F., Ito M., Li Y.R., Dedhia P.H., Zheng Y., Shao M., Gay D.L., Ramos R., Hsi T.C. (2017). Regeneration of fat cells from myofibroblasts during wound healing. Science.

[45] Franz A., Wood W., Martin P. (2018). Fat Body Cells Are Motile and Actively Migrate to Wounds to Drive Repair and Prevent Infection. Dev. Cell.

[46] Luo L., Liu M. (2016). Adipose tissue in control of metabolism. J. Endocrinol..

[47] Griffith J.F., Yeung D.K., Ahuja A.T., Choy C.W., Mei W.Y., Lam S.S., Lam T.P., Chen Z.Y., Leung P.C. (2009). A study of bone marrow and subcutaneous fatty acid composition in subjects of varying bone mineral density. Bone.

[48] Shockley K.R., Lazarenko O.P., Czernik P.J., Rosen C.J., Churchill G.A., Lecka-Czernik B. (2009). PPARgamma2 nuclear receptor controls multiple regulatory pathways of osteoblast differentiation from marrow mesenchymal stem cells. J. Cell. Biochem..

[49] Lecka-Czernik B. (2012). Marrow fat metabolism is linked to the systemic energy metabolism. Bone.

[50] Krings A., Rahman S., Huang S., Lu Y., Czernik P.J., Lecka-Czernik B. (2012). Bone marrow fat has brown adipose tissue characteristics, which are attenuated with aging and diabetes. Bone.

[51] Sulston R.J., Cawthorn W.P. (2016). Bone marrow adipose tissue as an endocrine organ: Close to the bone?. Horm. Mol. Biol. Clin. Invest..

[52] Cartwright M.J., Tchkonia T., Kirkland J.L. (2007). Aging in adipocytes: Potential impact of inherent, depot-specific mechanisms. Exp. Gerontol..

[53] Weisberg S.P., McCann D., Desai M., Rosenbaum M., Leibel R.L., Ferrante A.W. (2003). Obesity is associated with macrophage accumulation in adipose tissue. J. Clin. Invest..

[54] Xu H., Barnes G.T., Yang Q., Tan G., Yang D., Chou C.J., Sole J., Nichols A., Ross J.S., Tartaglia L.A. (2003). Chronic inflammation in fat plays a crucial role in the development of obesity-related insulin resistance. J. Clin. Invest..

[55] Dib L.H., Ortega M.T., Fleming S.D., Chapes S.K., Melgarejo T. (2014). Bone Marrow Leptin Signaling Mediates Obesity-Associated Adipose Tissue Inflammation in Male Mice. Endocrinology.

[56] Botolin S., McCabe L.R. (2007). Bone loss and increased bone adiposity in spontaneous and pharmacologically induced diabetic mice. Endocrinology.

[57] Ferland-McCollough D., Maselli D., Spinetti G., Sambataro M., Sullivan N., Blom A., Madeddu P. (2018). MCP-1 Feedback Loop Between Adipocytes and Mesenchymal Stromal Cells Causes Fat Accumulation and Contributes to Hematopoietic Stem Cell Rarefaction in the Bone Marrow of Patients With Diabetes. Diabetes.

[58] Bredella M.A., Fazeli P.K., Miller K.K., Misra M., Torriani M., Thomas B.J., Ghomi R.H., Rosen C.J., Klibanski A. (2009). Increased bone marrow fat in anorexia nervosa. J. Clin. Endocrinol. Metab..

[59] Devlin M.J., Cloutier A.M., Thomas N.A., Panus D.A., Lotinun S., Pinz I., Baron R., Rosen C.J., Bouxsein M.L. (2010). Caloric restriction leads to high marrow adiposity and low bone mass in growing mice. J. Bone Miner. Res..

[60] Turner R.T., Martin S.A., Iwaniec U.T. (2018). Metabolic Coupling Between Bone Marrow Adipose Tissue and Hematopoiesis. Curr. Osteoporos. Rep..

[61] Turner R.T., Wong C.P., Iwaniec U.T. (2011). Effect of Reduced c-Kit Signaling on Bone Marrow Adiposity. Anat. Rec..

[62] Mauriege P., De Pergola G., Berlan M., Lafontan M. (1988). Human fat cell beta-adrenergic receptors: Beta-agonist-dependent lipolytic responses and characterization of beta-adrenergic binding sites on human fat cell membranes with highly selective beta 1-antagonists. J. Lipid Res..

[63] Yoneshiro T., Shin W., Machida K., Fukano K., Tsubota A., Chen Y., Yasui H., Inanami O., Okamatsu-Ogura Y., Kimura K. (2019). Differentiation of bone marrow-derived cells toward thermogenic adipocytes in white adipose tissue induced by the beta 3 adrenergic stimulation. FASEB J..

[64] Mendez-Ferrer S., Lucas D., Battista M., Frenette P.S. (2008). Haematopoietic stem cell release is regulated by circadian oscillations. Nature.

[65] Arranz L., Sanchez-Aguilera A., Martin-Perez D., Isern J., Langa X., Tzankov A., Lundberg P., Muntion S., Tzeng Y.S., Lai D.M. (2014). Neuropathy of haematopoietic stem cell niche is essential for myeloproliferative neoplasms. Nature.

[66] Carriere A., Jeanson Y., Berger-Muller S., Andre M., Chenouard V., Arnaud E., Barreau C., Walther R., Galinier A., Wdziekonski B. (2014). Browning of white adipose cells by intermediate metabolites: An adaptive mechanism to alleviate redox pressure. Diabetes.

[67] Mills E.L., Pierce K.A., Jedrychowski M.P., Garrity R., Winther S., Vidoni S., Yoneshiro T., Spinelli J.B., Lu G.Z., Kazak L. (2018). Accumulation of succinate controls activation of adipose tissue thermogenesis. Nature.

[68] Guo Y., Xie C., Li X., Yang J., Yu T., Zhang R., Zhang T., Saxena D., Snyder M., Wu Y. (2017). Succinate and its G-protein-coupled receptor stimulates osteoclastogenesis. Nat. Commun..

[69] Grimolizzi F., Arranz L. (2018). Multiple faces of succinate beyond metabolism in blood. Haematologica.

[70] Kugel H., Jung C., Schulte O., Heindel W. (2001). Age- and sex-specific differences in the 1H-spectrum of vertebral bone marrow. J. Magn. Reson. Imaging.

[71] Griffith J.F., Yeung D.K., Ma H.T., Leung J.C., Kwok T.C., Leung P.C. (2012). Bone marrow fat content in the elderly: A reversal of sex difference seen in younger subjects. J. Magn. Reson. Imaging.

[72] Syed F.A., Oursler M.J., Hefferanm T.E., Peterson J.M., Riggs B.L., Khosla S. (2008). Effects of estrogen therapy on bone marrow adipocytes in postmenopausal osteoporotic women. Osteoporosis Int..

[73] McKerns K.W., Clynes R. (1961). Sex difference in rat adipose tissue metabolism. Metabolism.

[74] Zhang R., Su D., Zhu W., Huang Q., Liu M., Xue Y., Zhang Y., Li D., Zhao A., Liu Y. (2014). Estrogen suppresses adipogenesis by inhibiting S100A16 expression. J. Mol. Endocrinol..

[75] Jeffery E., Wing A., Holtrup B., Sebo Z., Kaplan J.L., Saavedra-Pena R., Church C.D., Colman L., Berry R., Rodeheffer M.S. (2016). The Adipose Tissue Microenvironment Regulates Depot-Specific Adipogenesis in Obesity. Cell Metab..

[76] White U.A., Tchoukalova Y.D. (2014). Sex dimorphism and depot differences in adipose tissue function. Biochim. Biophys. Acta.

[77] Ibrahim M.M. (2010). Subcutaneous and visceral adipose tissue: Structural and functional differences. Obes. Rev..

[78] Wajchenberg B.L. (2000). Subcutaneous and visceral adipose tissue: Their relation to the metabolic syndrome. Endocr. Rev..

[79] Bredella M.A., Torriani M., Ghomi R.H., Thomas B.J., Brick D.J., Gerweck A.V., Rosen C.J., Klibanski A., Miller K.K. (2011). Vertebral bone marrow fat is positively associated with visceral fat and inversely associated with IGF-1 in obese women. Obesity.

[80] Iwaniec U.T., Turner R.T. (2013). Failure to generate bone marrow adipocytes does not protect mice from ovariectomy-induced osteopenia. Bone.

[81] Sharp J.C., Copps J.C., Liu Q., Ryner L.N., Sebastian R.A., Zeng G.Q., Smith S., Niere J.O., Tomanek B., Sato M. (2000). Analysis of ovariectomy and estrogen effects on body composition in rats by X-ray and magnetic resonance imaging techniques. J. Bone Miner. Res..

[82] Nakada D., Oguro H., Levi B.P., Ryan N., Kitano A., Saitoh Y., Takeichi M., Wendt G.R., Morrison S.J. (2014). Oestrogen increases haematopoietic stem-cell self-renewal in females and during pregnancy. Nature.

[83] Sánchez-Aguilera A., Arranz L., Martín-Pérez D., García-García A., Stavropoulou V., Kubovcakova L., Isern J., Martín-Salamanca S., Langa X., Skoda R.C. (2014). Estrogen Signaling Selectively Induces Apoptosis of Hematopoietic Progenitors and Myeloid Neoplasms without Harming Steady-State Hematopoiesis. Cell Stem Cell.

[84] Kelly D.M., Jones T.H. (2015). Testosterone and obesity. Obes. Rev..

[85] Bianchi V.E., Locatelli V. (2018). Testosterone a key factor in gender related metabolic syndrome. Obes. Rev..

[86] Mitsuhashi K., Senmaru T., Fukuda T., Yamazaki M., Shinomiya K., Ueno M., Kinoshita S., Kitawaki J., Katsuyama M., Tsujikawa M. (2016). Testosterone stimulates glucose uptake and GLUT4 translocation through LKB1/AMPK signaling in 3T3-L1 adipocytes. Endocrine.

[87] Suzuki M., Murakami M., Shirai M., Hashimoto O., Kawada T., Matsui T., Funaba M. (2018). Role of estradiol and testosterone in Ucp1 expression in brown/beige adipocytes. Cell Biochem. Funct..

[88] Calvi L.M., Adams G.B., Weibrecht K.W., Weber J.M., Olson D.P., Knight M.C., Martin R.P., Schipani E., Divieti P., Bringhurst F.R. (2003). Osteoblastic cells regulate the haematopoietic stem cell niche. Nature.

[89] Zhang J., Niu C., Ye L., Huang H., He X., Tong W.G., Ross J., Haug J., Johnson T., Feng J.Q. (2003). Identification of the haematopoietic stem cell niche and control of the niche size. Nature.

[90] Kiel M.J., Yilmaz O.H., Iwashita T., Yilmaz O.H., Terhorst C., Morrison S.J. (2005). SLAM family receptors distinguish hematopoietic stem and progenitor cells and reveal endothelial niches for stem cells. Cell.

[91] Acar M., Kocherlakota K.S., Murphy M.M., Peyer J.G., Oguro H., Inra C.N., Jaiyeola C., Zhao Z., Luby-Phelps K., Morrison S.J. (2015). Deep imaging of bone marrow shows non-dividing stem cells are mainly perisinusoidal. Nature.

[92] Ding L., Saunders T.L., Enikolopov G., Morrison S.J. (2012). Endothelial and perivascular cells maintain haematopoietic stem cells. Nature.

[93] Sugiyama T., Kohara H., Noda M., Nagasawa T. (2006). Maintenance of the hematopoietic stem cell pool by CXCL12-CXCR4 chemokine signaling in bone marrow stromal cell niches. Immunity.

[94] Ding L., Morrison S.J. (2013). Haematopoietic stem cells and early lymphoid progenitors occupy distinct bone marrow niches. Nature.

[95] Gomes A.C., Hara T., Lim V.Y., Herndler-Brandstetter D., Nevius E., Sugiyama T., Tani-ichi S., Schlenner S., Richie E., Rodewald H.R. (2016). Hematopoietic Stem Cell Niches Produce Lineage-Instructive Signals to Control Multipotent Progenitor Differentiation. Immunity.

[96] Balzano M., De Grandis M., Vu Manh T.P., Chasson L., Bardin F., Farina A., Serge A., Bidaut G., Charbord P., Herault L. (2019). Nidogen-1 Contributes to the Interaction Network Involved in Pro-B Cell Retention in the Peri-sinusoidal Hematopoietic Stem Cell Niche. Cell Rep..

[97] Greenbaum A., Hsu Y.M., Day R.B., Schuettpelz L.G., Christopher M.J., Borgerding J.N., Nagasawa T., Link D.C. (2013). CXCL12 in early mesenchymal progenitors is required for haematopoietic stem-cell maintenance. Nature.

[98] Kfoury Y., Scadden D.T. (2015). Mesenchymal cell contributions to the stem cell niche. Cell Stem Cell.

[99] Gordon M.Y. (1994). Stem cells and the microenvironment in aplastic anaemia. Br. J. Haematol..

[100] Zhu R.J., Wu M.Q., Li Z.J., Zhang Y., Liu K.Y. (2013). Hematopoietic recovery following chemotherapy is improved by BADGE-induced inhibition of adipogenesis. Int. J. Hematol..

[101] Yamazaki K., Allen T.D. (1991). Ultrastructural and morphometric alterations in bone marrow stromal tissue after 7 Gy irradiation. Blood Cells.

[102] DiMascio L., Voermans C., Uqoezwa M., Duncan A., Lu D., Wu J., Sankar U., Reya T. (2007). Identification of adiponectin as a novel hemopoietic stem cell growth factor. J. Immunol..

[103] Masamoto Y., Arai S., Sato T., Kubota N., Takamoto I., Kadowaki T., Kurokawa M. (2017). Adiponectin Enhances Quiescence Exit of Murine Hematopoietic Stem Cells and Hematopoietic Recovery Through mTORC1 Potentiation. Stem Cells.

[104] Yokota T., Oritani K., Takahashi I., Ishikawa J., Matsuyama A., Ouchi N., Kihara S., Funahashi T., Tenner A.J., Tomiyama Y. (2000). Adiponectin, a new member of the family of soluble defense collagens, negatively regulates the growth of myelomonocytic progenitors and the functions of macrophages. Blood.

[105] Yokota T., Meka C.S., Kouro T., Medina K.L., Igarashi H., Takahashi M., Oritani K., Funahashi T., Tomiyama Y., Matsuzawa Y. (2003). Adiponectin, a fat cell product, influences the earliest lymphocyte precursors in bone marrow cultures by activation of the cyclooxygenase-prostaglandin pathway in stromal cells. J. Immunol..

[106] Yokota T., Meka C.S., Medina K.L., Igarashi H., Comp P.C., Takahashi M., Nishida M., Oritani K., Miyagawa J., Funahashi T. (2002). Paracrine regulation of fat cell formation in bone marrow cultures via adiponectin and prostaglandins. J. Clin. Invest..

[107] Ito K., Carracedo A., Weiss D., Arai F., Ala U., Avigan D.E., Schafer Z.T., Evans R.M., Suda T., Lee C.H. (2012). A PML-PPAR-delta pathway for fatty acid oxidation regulates hematopoietic stem cell maintenance. Nat. Med..

[108] Claycombe K., King L.E., Fraker P.J. (2008). A role for leptin in sustaining lymphopoiesis and myelopoiesis. Proc. Natl. Acad. Sci. USA.

[109] Pietramaggiori G., Scherer S.S., Alperovich M., Chen B., Orgill D.P., Wagers A.J. (2009). Improved Cutaneous Healing in Diabetic Mice Exposed to Healthy Peripheral Circulation. J. Invest. Dermatol..

[110] Wang L.D., Wagers A.J. (2011). Dynamic niches in the origination and differentiation of haematopoietic stem cells. Nat. Rev. Mol. Cell Bio..

[111] Laharrague P., Larrouy D., Fontanilles A.M., Truel N., Campfield A., Tenenbaum R., Galitzky J., Corberand J.X., Penicaud L., Casteilla L. (1998). High expression of leptin by human bone marrow adipocytes in primary culture. FASEB J..

[112] Zhang Y., Proenca R., Maffei M., Barone M., Leopold L., Friedman J.M. (1994). Positional cloning of the mouse obese gene and its human homologue. Nature.

[113] Friedman J.M., Halaas J.L. (1998). Leptin and the regulation of body weight in mammals. Nature.

[114] Giralt M., Cereijo R., Villarroya F. (2016). Adipokines and the Endocrine Role of Adipose Tissues. Handb. Exp. Pharmacol..

[115] Faggioni R., Jones-Carson J., Reed D.A., Dinarello C.A., Feingold K.R., Grunfeld C., Fantuzzi G. (2000). Leptin-deficient (ob/ob) mice are protected from T cell-mediated hepatotoxicity: Role of tumor necrosis factor alpha and IL-18. Proc. Natl. Acad. Sci. USA.

[116] Bennett B.D., Solar G.P., Yuan J.Q., Mathias J., Thomas G.R., Matthews W. (1996). A role for leptin and its cognate receptor in hematopoiesis. Curr. Biol..

[117] Fantuzzi G., Faggioni R. (2000). Leptin in the regulation of immunity, inflammation, and hematopoiesis. J. Leukocyte Biol..

[118] Gainsford T., Willson T.A., Metcalf D., Handman E., McFarlane C., Ng A., Nicola N.A., Alexander W.S., Hilton D.J. (1996). Leptin can induce proliferation, differentiation, and functional activation of hemopoietic cells. Proc. Natl. Acad. Sci. USA.

[119] Umemoto Y., Tsuji K., Yang F.C., Ebihara Y., Kaneko A., Furukawa S., Nakahata T. (1997). Leptin stimulates the proliferation of murine myelocytic and primitive hematopoietic progenitor cells. Blood.

[120] Yue R., Zhou B.O., Shimada I.S., Zhao Z., Morrison S.J. (2016). Leptin Receptor Promotes Adipogenesis and Reduces Osteogenesis by Regulating Mesenchymal Stromal Cells in Adult Bone Marrow. Cell Stem Cell.

[121] Chen L., Gao Z., Zhu J., Rodgers G.P. (2007). Identification of CD13+CD36+ cells as a common progenitor for erythroid and myeloid lineages in human bone marrow. Exp. Hematol..

[122] Dirat B., Bochet L., Dabek M., Daviaud D., Dauvillier S., Majed B., Wang Y.Y., Meulle A., Salles B., Le Gonidec S. (2011). Cancer-Associated Adipocytes Exhibit an Activated Phenotype and Contribute to Breast Cancer Invasion. Cancer Res..

[123] Laharrague P., Fontanilles A.M., Tkaczuk J., Corberand J.X., Penicaud L., Casteilla L. (2000). Inflammatory/haematopoietic cytokine production by human bone marrow adipocytes. Eur. Cytok. Netw..

[124] Mattiucci D., Maurizi G., Izzi V., Cenci L., Ciarlantini M., Mancini S., Mensa E., Pascarella R., Vivarelli M., Olivieri A. (2018). Bone marrow adipocytes support hematopoietic stem cell survival. J. Cell. Physiol..

[125] Spindler T.J., Tseng A.W., Zhou X., Adams G.B. (2014). Adipocytic cells augment the support of primitive hematopoietic cells in vitro but have no effect in the bone marrow niche under homeostatic conditions. Stem Cells Dev..

[126] Corre J., Planat-Benard V., Corberand J.X., Penicaud L., Casteilla L., Laharrague P. (2004). Human bone marrow adipocytes support complete myeloid and lymphoid differentiation from human CD34 cells. Br. J. Hematol..

[127] Glettig D.L., Kaplan D.L. (2013). Extending human hematopoietic stem cell survival in vitro with adipocytes. BioRes. Open Access.

[128] Belaid-Choucair Z., Lepelletier Y., Poncin G., Thiry A., Humblet C., Maachi M., Beaulieu A., Schneider E., Briquet A., Mineur P. (2008). Human bone marrow adipocytes block granulopoiesis through neuropilin-1-induced granulocyte colony-stimulating factor inhibition. Stem Cells.

[129] Raaijmakers M.H., Mukherjee S., Guo S., Zhang S., Kobayashi T., Schoonmaker J.A., Ebert B.L., Al-Shahrour F., Hasserjian R.P., Scadden E.O. (2010). Bone progenitor dysfunction induces myelodysplasia and secondary leukaemia. Nature.

[130] Dong L., Yu W.M., Zheng H., Loh M.L., Bunting S.T., Pauly M., Huang G., Zhou M.X., Broxmeyer H.E., Scadden D.T. (2016). Leukaemogenic effects of Ptpn11 activating mutations in the stem cell microenvironment. Nature.

[131] Walkley C.R., Olsen G.H., Dworkin S., Fabb S.A., Swann J., McArthur G.A., Westmoreland S.V., Chambon P., Scadden D.T., Purton L.E. (2007). A microenvironment-induced myeloproliferative syndrome caused by retinoic acid receptor gamma deficiency. Cell.

[132] Kode A., Manavalan J.S., Mosialou I., Bhagat G., Rathinam C.V., Luo N., Khiabanian H., Lee A., Murty V.V., Friedman R. (2014). Leukaemogenesis induced by an activating beta-catenin mutation in osteoblasts. Nature.

[133] Sanchez-Aguilera A., Mendez-Ferrer S. (2017). The hematopoietic stem-cell niche in health and leukemia. Cell Mol. Life Sci..

[134] Warburg O. (1956). On the origin of cancer cells. Science.

[135] Carracedo A., Cantley L.C., Pandolfi P.P. (2013). Cancer metabolism: Fatty acid oxidation in the limelight. Nat. Rev. Cancer.

[136] Jones R.G., Thompson C.B. (2009). Tumor suppressors and cell metabolism: A recipe for cancer growth. Genes Dev..

[137] Ye H., Adane B., Khan N., Sullivan T., Minhajuddin M., Gasparetto M., Stevens B., Pei S., Balys M., Ashton J.M. (2016). Leukemic Stem Cells Evade Chemotherapy by Metabolic Adaptation to an Adipose Tissue Niche. Cell Stem Cell.

[138] Yan F., Shen N., Pang J.X., Zhang Y.W., Rao E.Y., Bode A.M., Al-Kali A., Zhang D.E., Litzow M.R., Li B. (2017). Fatty acid-binding protein FABP4 mechanistically links obesity with aggressive AML by enhancing aberrant DNA methylation in AML cells. Leukemia.

[139] Shafat M.S., Oellerich T., Mohr S., Robinson S.D., Edwards D.R., Marlein C.R., Piddock R.E., Fenech M., Zaitseva L., Abdul-Aziz A. (2017). Leukemic blasts program bone marrow adipocytes to generate a protumoral microenvironment. Blood.

[140] Samudio I., Harmancey R., Fiegl M., Kantarjian H., Konopleva M., Korchin B., Kaluarachchi K., Bornmann W., Duvvuri S., Taegtmeyer H. (2010). Pharmacologic inhibition of fatty acid oxidation sensitizes human leukemia cells to apoptosis induction. J. Clin. Invest..

[141] Tung S., Shi Y., Wong K., Zhu F., Gorczynski R., Laister R.C., Minden M., Blechert A.K., Genzel Y., Reichl U. (2013). PPARalpha and fatty acid oxidation mediate glucocorticoid resistance in chronic lymphocytic leukemia. Blood.

[142] Prost S., Relouzat F., Spentchian M., Ouzegdouh Y., Saliba J., Massonnet G., Beressi J.P., Verhoeyen E., Raggueneau V., Maneglier B. (2015). Erosion of the chronic myeloid leukaemia stem cell pool by PPARgamma agonists. Nature.

[143] Castillo J.J., Reagan J.L., Ingham R.R., Furman M., Dalia S., Merhi B., Nemr S., Zarrabi A., Mitri J. (2012). Obesity but not overweight increases the incidence and mortality of leukemia in adults: A meta-analysis of prospective cohort studies. Leukemia Res..

[144] Tabe Y., Yamamoto S., Saitoh K., Sekihara K., Monma N., Ikeo K., Mogushi K., Shikami M., Ruvolo V., Ishizawa J. (2017). Bone Marrow Adipocytes Facilitate Fatty Acid Oxidation Activating AMPK and a Transcriptional Network Supporting Survival of Acute Monocytic Leukemia Cells. Cancer Res..

[145] Lu W., Wan Y., Li Z., Zhu B., Yin C., Liu H., Yang S., Zhai Y., Yu Y., Wei Y. (2018). Growth differentiation factor 15 contributes to marrow adipocyte remodeling in response to the growth of leukemic cells. J. Exp. Clin. Cancer Res..

[146] Willson T.M., Cobb J.E., Cowan D.J., Wiethe R.W., Correa I.D., Prakash S.R., Beck K.D., Moore L.B., Kliewer S.A., Lehmann J.M. (1996). The structure-activity relationship between peroxisome proliferator-activated receptor gamma agonism and the antihyperglycemic activity of thiazolidinediones. J. Med. Chem..

[147] Hu W., Jiang C., Guan D., Dierickx P., Zhang R., Moscati A., Nadkarni G.N., Steger D.J., Loos R.J.F., Hu C. (2019). Patient Adipose Stem Cell-Derived Adipocytes Reveal Genetic Variation that Predicts Antidiabetic Drug Response. Cell Stem Cell.

[148] Samimi A., Ghanavat M., Shahrabi S., Azizidoost S., Saki N. (2019). Role of bone marrow adipocytes in leukemia and chemotherapy challenges. Cell Mol. Life Sci..

[149] Han T.J., Wang X. (2015). Leptin and its receptor in hematologic malignancies. Int. J. Clin. Exp. Med..

[150] Tabe Y., Konopleva M., Munsell M.F., Marini F.C., Zompetta C., McQueen T., Tsao T., Zhao S., Pierce S., Igari J. (2004). PML-RARalpha is associated with leptin-receptor induction: The role of mesenchymal stem cell-derived adipocytes in APL cell survival. Blood.

[151] Caers J., Deleu S., Belaid Z., De Raeve H., Van Valckenborgh E., De Bruyne E., Defresne M.P., Van Riet I., Van Camp B., Vanderkerken K. (2007). Neighboring adipocytes participate in the bone marrow microenvironment of multiple myeloma cells. Leukemia.

[152] Favreau M., Menu E., Gaublomme D., Vanderkerken K., Faict S., Maes K., De Bruyne E., Govindarajan S., Drennan M., Van Calenbergh S. (2017). Leptin receptor antagonism of iNKT cell function: A novel strategy to combat multiple myeloma. Leukemia.

[153] Piya M.K., McTernan P.G., Kumar S. (2013). Adipokine inflammation and insulin resistance: The role of glucose, lipids and endotoxin. J. Endocrinol..

[154] Yamauchi T., Kadowaki T. (2013). Adiponectin Receptor as a Key Player in Healthy Longevity and Obesity-Related Diseases. Cell Metab..

[155] Medina E.A., Oberheu K., Polusani S.R., Ortega V., Velagaleti G.V.N., Oyajobi B.O. (2014). PKA/AMPK signaling in relation to adiponectin’s antiproliferative effect on multiple myeloma cells. Leukemia.

[156] Cahu X., Calvo J., Poglio S., Prade N., Colsch B., Arcangeli M.L., Leblanc T., Petit A., Baleydier F., Baruchel A. (2017). Bone marrow sites differently imprint dormancy and chemoresistance to T-cell acute lymphoblastic leukemia. Blood Adv..

[157] Behan J.W., Avramis V.I., Yun J.P., Louie S.G., Mittelman S.D. (2010). Diet-induced obesity alters vincristine pharmacokinetics in blood and tissues of mice. Pharmacol. Res..

[158] Behan J.W., Yun J.P., Proektor M.P., Ehsanipour E.A., Arutyunyan A., Moses A.S., Avramis V.I., Louie S.G., Butturini A., Heisterkamp N. (2009). Adipocytes Impair Leukemia Treatment in Mice. Cancer Res..

[159] Pramanik R., Sheng X., Ichihara B., Heisterkamp N., Mittelman S.D. (2013). Adipose tissue attracts and protects acute lymphoblastic leukemia cells from chemotherapy. Leukemia Res..

[160] Sheng X., Parmentier J.H., Tucci J., Pei H., Cortez-Toledo O., Dieli-Conwright C.M., Oberley M.J., Neely M., Orgel E., Louie S.G. (2017). Adipocytes Sequester and Metabolize the Chemotherapeutic Daunorubicin. Mol. Cancer Res..

[161] Liu H., Zhai Y., Zhao W., Wan Y., Lu W., Yang S., Yu Y., Wei Y., Li Z., Shi J. (2018). Consolidation Chemotherapy Prevents Relapse by Indirectly Regulating Bone Marrow Adipogenesis in Patients with Acute Myeloid Leukemia. Cell Physiol. Biochem..

[162] Konieczny J., Arranz L. (2018). Updates on Old and Weary Haematopoiesis. Int. J. Mol. Sci..

[163] Luche E., Robert V., Cuminetti V., Pomie C., Sastourne-Arrey Q., Waget A., Arnaud E., Varin A., Labit E., Laharrague P. (2017). Corrupted adipose tissue endogenous myelopoiesis initiates diet-induced metabolic disease. eLife.

[164] Poglio S., De Toni F., Lewandowski D., Minot A., Arnaud E., Barroca V., Laharrague P., Casteilla L., Cousin B. (2012). In situ production of innate immune cells in murine white adipose tissue. Blood.

